# Blueprints for Change: Integrating Systems Thinking into Musculoskeletal Health Policy; A Response to Recent Commentaries

**DOI:** 10.34172/ijhpm.9387

**Published:** 2025-09-14

**Authors:** Carmen Huckel Schneider, Helen Slater, Deborah Kopansky-Giles, Lyn March, Sarika Parambath, Saurab Sharma, James J. Young, Swatee Jain, Andrew M. Briggs

**Affiliations:** ^1^Leeder Centre for Health Policy, Economics and Data, Faculty of Medicine and Health, University of Sydney, Sydney, NSW, Australia.; ^2^Curtin School of Allied Health, and enAble Institute, Faculty of Health Sciences, Curtin University, Perth, WA, Australia.; ^3^Department of Research, Canadian Memorial Chiropractic College, Toronto, ON, Canada.; ^4^Department of Family & Community Medicine, University of Toronto, Toronto, ON, Canada.; ^5^Florance and Cope Professorial Department of Rheumatology, Royal North Shore Hospital and Kolling Institute, University of Sydney, Sydney, NSW, Australia.; ^6^Kolling Institute, Faculty of Medicine and Health, University of Sydney, Sydney, NSW, Australia.; ^7^Center for Muscle and Joint Health, Faculty of Health Sciences, University of Southern Denmark, Odense, Denmark.

 We thank Deane^1^ and Lall^2^ for their insightful commentaries on our recent article.^3^ Their reflections underscore the importance of advancing musculoskeletal (MSK) health within global health policy and systems reform initiatives, within Agenda 2030, and beyond. The commentaries provide valuable perspectives that highlight an urgency to deepen our understanding of structures and systems for improved advocacy and evidence-based policy for MSK health globally. Critically, the comments urge for transitioning from policy and systems guidance to implementation efforts within countries.

 In synthesizing their contributions alongside our own findings, we identify three interlocking themes that are central to progressing health policy globally that is inclusive of MSK health. First, both commentaries highlight the need to elevate MSK health as a priority within the global policy agenda for non-communicable diseases (NCDs). As Lall notes,^2^ despite their significant contribution to disability and health system burden (eg, service demand, cost, workforce), MSK conditions are often underrepresented in policy and essential care packages, such as universal health coverage. Our work supports the call for MSK health to be more prominently integrated into universal health coverage strategies for NCDs, rehabilitation and health and well-being across the life course.

 Second, Deane’s framing of MSK health impairments as a “wicked problem” is particularly relevant to systems strengthening initiatives.^1^ The complex aetiology and multifactorial nature of MSK conditions—spanning structural health system components and broader social, political, cultural, environmental, and commercial determinants—require systems thinking and adaptive policy responses, all reflective of our proposed blueprint for strengthening health systems.^4^ Both commentaries emphasize the need for dynamic, whole-of-system approaches that recognise the interconnected realities of complex health ecosystems. In this context, we appreciate the debate on the utility of the World Health Organization (WHO) Building Blocks model. We applied this model in our work because it is well-known and fit-for-purpose to organise our inductively derived findings. Consistent with others’ views,^5^ our findings identify the limitations of this model and gaps in national policy formulation, including an inadequate focus on equity, community participation and lived experience. Importantly, when interpreted in a static way, the model does not depict the dynamic relationships and interdependencies between parts of the health ecosystem (the Blocks), which are critical to understanding complex system function. We agree with the commentators that future policy must evolve beyond foundational components (single Blocks) to embrace the dynamic and interconnected nature of continuously evolving health systems. Here, Deanne’s proposal for the application of complexity theory models represents one approach and we welcome the further development of frameworks suitable to global health systems strengthening efforts.^1^

 Third, achieving integrated care that is inclusive of MSK health, demands more than structural reform in policy, workforce, service models, and financing; it requires a paradigm shift in how care is conceptualized and delivered across the life course.^6^ Here, a commitment to prevention and control of co-and multi-multimorbidity that includes MSK health is essential, while also supporting the empowerment of community participation and strengthening primary care capacity and services for MSK care.^4^

 Our work began prior to the COVID-19 pandemic, building on the call made in our 2019 article for system- and service-level responses to the global burden of MSK pain.^7^ We highlighted that this burden persists across high-, middle-, and low-income settings, yet remains mismatched with health policy responses and planning. The latter focussed on identifying the scale of the problem, rather than avenues to build strong solutions. We argued then—and continue to emphasize now—that this gap can be addressed through an integrated research and policy agenda. MSK health and pain must be explicitly recognized in policy, not in isolation, but as part of a cohesive strategy alongside other NCDs in a life course and equity-informed approach. This is important, now more than ever, in the context of the Fourth High-Level Meeting of the United Nations General Assembly on the Prevention and Control of NCDs.

 Our study^3^ was part of a broader program of work grounded in an understanding of complexity and aimed at progressing global MSK health policy.^4^ The comparative policy content analysis used a grounded approach, drawing themes from empirical realities at the national level. Surrounding this, we undertook two additional phases. First, we conducted key informant interviews with 31 individuals from 25 organizations across 20 countries, 40% of which were low- and middle-income countries. We identified a logic model comprising five guiding principles, eight strategic priority areas (pillars), and seven accelerators for action. We found these aligned closely with the themes that emerged in our policy content analysis.^3^ Second, we implemented a global eDelphi process involving over 650 panellists from 72 countries (46% low- and middle-income countries). This process enabled multisectoral experts to iterate and prioritise detailed actions underpinning each pillar (Figure).

**Figure:**
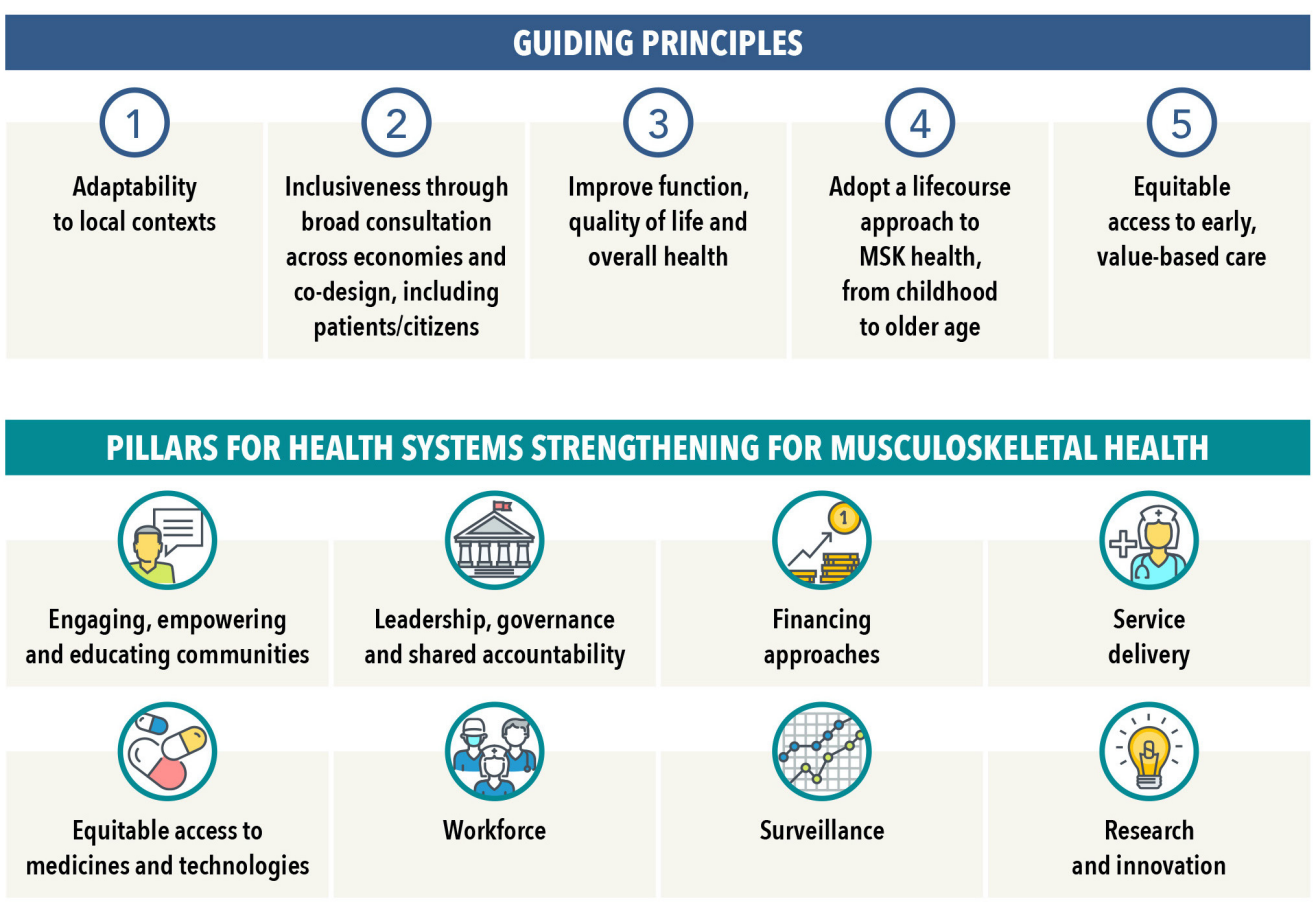
Overview of the Guiding Principles and Pillars for Action to Strengthen Health Systems to Address Prevention and Control of Musculoskeletal Health. Abbreviation: MSK, musculoskeletal. Adapted from Briggs et al^4^ under Creative Commons Attribution Non-Commercial (CC BY-NC 4.0).

 The eight pillars and their components capture the breadth of health systems strengthening at both the macro (whole-of-system) and meso (service delivery/organisational) levels. This scope aligns with broader system transformation priorities within Agenda 2030 that overlap MSK health, such as healthy ageing and rehabilitation. While the eight pillars closely correspond with established frameworks, including the WHO Health Systems Building Blocks, they also reflect models of value-driven learning health systems. When considered alongside findings from our policy review, the inter-model alignment supports the construct validity of our logic model and enhances its relevance and usability for policy-makers and stakeholders, as identified in a recent evaluation.^8^

 The resulting empirically-derived framework and data-derived logic model offers a blueprint for global and country-level responses to strengthen health systems for improved MSK health. We see this work as a set of entry points and opportunities to address the wicked problems inherent in MSK health policy and global health. In moving towards the conclusion of Agenda 2030 and the start of renewed global commitments to health, supporting countries to evolve national and sub-national health policy inclusive of MSK health, and to implement inclusive service models, will be essential to arresting the increasing attributable global burden of disease.

## Ethical issues

 Not applicable.

## Conflicts of interest

 Authors declare that they have no conflicts of interest.
